# Maguey, or Agave Americana, as a Remedy for Scorbutus

**Published:** 1851-11

**Authors:** 


*Maguey, or Agave Americana, as a Remedy for Scorbutus.*—The expressed juice of the Agave Americana, the American Aloe or Maguey, as it is termed in Mexico, has lately been used with the greatest success among the troops at Fort McIntosh, in Texas, as a remedy for Scorbutus. Dr. G. Perin, Assistant Surgeon U. S. A., reports several cases treated with the Maguey, which go to show it to be greatly superior to other remedies in the disease in question. A notice of the Maguey is to be found among the unofficinal articles in the U. S. Dispensatory. It is indigenous in Texas, and, Dr. Perin thinks, also in New Mexico and California. In Mexico, it grows in great abundance, and is the plant from which the favorite drink, "pulque," is made.

"The manner in which it is used is as follows, viz.:—The leaves are cut off close to the root, they are placed in hot ashes until thoroughly cooked, when they are removed, and the juice expressed from them. The expressed juice is then strained, and may be used thus, or may be sweetened. It may be given in doses of f.℥ij. to f.℥iij. three times daily.

It is not disagreeable to take, and in every instance it has proved to agree well with the stomach and bowels.

After the leaves have been cooked, the cortical portion near the root may be removed, and the white internal portion may be eaten; it appears to be a wholesome and nutritious food. Dr. P. has seen muleteers use it in this way, and they seem to be very fond of it. He has been informed, upon good authority, that several tribes of Indians in New Mexico make use of it in the same manner. The use of the leaf in this way, he believes, will ward off most effectually incipient scorbutus." —*Abridged from N. Y. Journal of Medicine*, September 1851.

